# Photoacoustic imaging reconstruction using combined nonlocal patch and total-variation regularization for straight-line scanning

**DOI:** 10.1186/s12938-018-0537-x

**Published:** 2018-08-03

**Authors:** Jin Wang, Yuanyuan Wang

**Affiliations:** 1 Department of Electronic Engineering, Fudan University, No. 220 Handan Road, Shanghai, 200433 China; 2 Key Laboratory of Medical Imaging Computing and Computer Assisted Intervention (MICCAI) of Shanghai, Fudan University, Shanghai, 200433 China

**Keywords:** Photoacoustic imaging, Straight-line reconstruction, Nonlocal patch, Total variation

## Abstract

**Background:**

For practical straight-line scanning in photoacoustic imaging (PAI), serious artifacts caused by missing data will occur. Traditional total variation (TV)-based algorithms fail to obtain satisfactory results, with an over-smoothed and blurred geometric structure. Therefore, it is important to develop a new algorithm to improve the quality of practical straight-line reconstructed images.

**Methods:**

In this paper, a combined nonlocal patch and TV-based regularization model for PAI reconstruction is proposed to solve these problems. A modified adaptive nonlocal weight function is adopted to provide more reliable estimations for the similarities between patches. Similar patches are searched for throughout the entire image; thus, this model realizes adaptive search for the neighborhood of the patch. The optimization problem is simplified to a common iterative PAI reconstruction problem.

**Results and conclusion:**

The proposed algorithm is validated by a series of numerical simulations and an in vitro experiment for straight-line scanning. The results of patch-TV are compared to those of two mainstream TV-based algorithms as well as the iterative algorithm only with patch-based regularization. Moreover, the peak signal-to-noise ratio, the noise robustness, and the convergence and calculation speeds are compared and discussed. The results show that the proposed patch-TV yields significant improvement over the other three algorithms qualitatively and quantitatively. These simulations and experiment indicate that the patch-TV algorithm successfully solves the problems of PAI reconstruction and is highly effective in practical PAI applications.

## Background

Photoacoustic imaging (PAI), a novel biomedical imaging technique, combines light and ultrasound to detect absorbed photons ultrasonically through the photoacoustic effect [1–3]. Compared with traditional imaging techniques, PAI has many advantages. It obtains high image contrast because the photoacoustic images can reflect the laser absorption distribution in the tissue [1]. It is capable of imaging either thicker tissue or deeper organs with better resolution compared to optical imaging because it receives ultrasound signals [3]. What’s more, PAI is also able to provide noninvasive and functional imaging [4, 5]. Because of these advantages, PAI shows great potential in many biomedical applications such as brain imaging [6, 7], tumor detection [8, 9], vessel imaging [10, 11] and molecular imaging [12, 13].

A laser pulse is usually adopted to irradiate the tissue in computed-tomographic PAI, which is the main concern of this paper. The light is absorbed by the tissue, and the ultrasound waves are subsequently excited. This process is called the photoacoustic effect [1]. Then, the photoacoustic signals are detected by a scanning transducer or a transducer array. To reconstruct the photoacoustic image from the detected signals, photoacoustic reconstruction algorithms are required, which directly determine the image quality of the reconstruction. Therefore, photoacoustic reconstruction algorithms play an essential role in computed-tomographic PAI.

Many efforts have been made to develop photoacoustic reconstruction algorithms. Analytical reconstruction algorithms were first developed, and their techniques are relatively mature [14–18]. The filtered back-projection (FBP) method proposed by Xu et al. was widely used due to its concision and convenience [16]. Zhang et al. proposed the deconvolution reconstruction algorithm, which achieved improved results in the case of both full-view and limited-view scanning [18]. To overcome the strong data dependency of the analytical reconstruction algorithms and improve their performance, the iterative image reconstruction methods were proposed. This kind of reconstruction methods established a forward model from photoacoustic image to photoacoustic signals to calculate the photoacoustic image iteratively [19–25]. Compressed sensing (CS) theory has been adopted in PAI to reduce the number of samples required and improve the results in sparse-view scanning [26–31]. Among these algorithms, total-variation (TV)-based reconstruction algorithms have achieved excellent reconstruction quality [32–38]. The TV minimization can greatly reduce the dependence on data so that images can be accurately recovered from sparse data. Therefore it is potential to improve the performance of the algorithm on limited-view scanning based on TV-method. An adaptive steepest-descent-projection onto convex sets (ASD-POCS) is proposed by Wang et al. to employ the TV-based iterative image reconstruction algorithms in three-dimensional PAI [33]. Zhang et al. proposed a gradient descent-based TV (TV-GD) algorithm, which was able to maintain good performance even in sparse-view scanning [34]. A joint TV and Lp-norm (TV-Lp)-based algorithm proposed by Zhang et al. was reported to have improved performance especially in the sparse-view scanning [39]. Besides, wavelets transform domain [21, 40], total generalised variation [41] as well as deep learning regularization [42, 43] have also been adopted in PAI reconstruction and reported to have successfully addressed some specific problems in PAI. While for wavelets transform domain [21, 40] as well as total generalised variation [41]—based method, there still exists room for improvement in the preservation of structure and detail information particularly under the circumstance of limited-view scanning. As for deep learning based methods [42, 43], the algorithms are too complex and difficult to implement.

The image reconstruction methods at the present stage have worked well with full-view sampled data, but in practical situations, full-view scanning is often unavailable because of the restraint of the body shape or firmware. Under such circumstances, only limited-view projection data can be acquired, which do not conform to the condition of data completeness. In biomedical clinical practice, the linear transducer array is one of the popular ways to collect ultrasound signals. For clinical application, current PAI reconstruction algorithms still have many problems, such as edge blur and serious artifacts [28, 30, 37, 38, 44–49]. There is still much room for improvement. It is necessary to develop an image reconstruction method that is effective in clinical applications.

The TV expresses local intensity changes in an image. The classical TV-based reconstruction methods were established based on the assumption that the images are piece-wise constant [50]. While the TV model has obtained a good effect in terms of sparse-view reconstruction, due to the over-inhibition of the high-frequency coefficients, minimizing the TV of an image tends to create over-smoothed geometry construction in the images [50–52]. The result is even worse in the case of practical limited-view scanning when some angular projection data are missing, as severe artifacts emerge and detailed information is lost [34, 37, 39]. In recent years, a nonlocal idea involving a priori knowledge that reveals the self-similarity of images has been proposed and widely used in image processing and reconstruction [53–56]. Minimizing TV can be regarded as minimizing the variation between adjacent pixels and can therefore be named local TV. Nonlocal TV extends the spatial neighborhood in the traditional neighborhood filtering to the structured neighborhood with a more generalized geometric meaning [56]. It searches similar patches in a larger area and uses the similarity between patches as the weight. This approach overcomes the limitation of traditional neighborhood weighting and makes better use of the similarities within images. Therefore, the reconstructed images can be improved in terms of texture and structure preservation. By solving the research and clinical problems, the method has obtained better performance in local TV [56–58].

In this paper, we propose a novel PAI reconstruction algorithm that incorporates nonlocal patch-based regularization into the TV-based model (patch-TV) to improve the reconstruction results for practical straight-line scanning. The patch in the image is estimated by weighting the patches in its neighborhood, which are searched throughout the whole image adaptively. The reconstructed image is updated by joint TV and nonlocal-patch regularization. The modified weighting calculation method is adopted with directivity and adaptability to further improve the performance of structure maintenance for the image [59]. Finally, the optimization model is simplified, and efficient variable splitting and the Barzilai–Borwein-based method are adopted to solve the optimization problem [60]. A series of numerical simulations and an in vitro experiment are conducted to validate the proposed patch-TV algorithm. The results of the patch-TV algorithm are compared to those of TV-based algorithms solved by the gradient descent method (TV-GD), the TV-Lp algorithm as well as the iterative algorithm only with patch-based regularization (Patch-RE). The peak signal-to-noise ratios (PSNRs), the noise robustness, and the calculation and convergence speeds are also discussed and compared. Both qualitative and quantitative comparisons show that the patch-TV algorithm provides superior results to those of TV-GD, TV-Lp and Patch-RE. The geometric structures of the images are preserved well, and the quality of the reconstructed images is greatly improved for practical straight-line scanning. A series of patch based methods have been applied in imaging, such as [61]. In [61], nonlocal patch was used as a filter to process the image after the updating of each iteration step, which makes the algorithm one kind of image processing rather than image reconstruction. Moreover, the simple and isotropic distance between two blocks is utilized to screen the neighborhood of the block. In the proposed patch-TV algorithm, non-local patch is used as a constraint item in the optimization problem for reconstruction. The optimization problem is then simplified to a common iterative PAI reconstruction problem so that the complexity of the algorithm is greatly reduced. The modified weighting calculation method which utilizes the modified structure tensor matrix to construct the weight function between two patches with directivity and adaptability is adopts in the proposed algorithm. The screened neighborhood of the patches takes the directivities and geometric structure of the images fully into consideration. It further improves the performance of structure preserving for the image. The nonlocal-patch regularization is combined with TV minimization in the proposed algorithm to obtain better performance in straight-line scanning with stability.

There are mainly three points for the contributions of this paper. First, we include the non-local patch idea into PAI reconstruction. As far as we know, it is the first time that non-local patch ideal is applied to PAI. Second, the combination of the non-local patch optimization and TV minimization has been firstly applied into PAI. This combined method is able to solve the problems of PAI reconstruction from straight-line scanning. Finally, we simplify the complicated optimization problem to a common iterative PAI reconstruction problem and use efficient variable splitting and the Barzilai–Borwein-based method to solve this problem. The optimization steps are greatly simplified and the convergence is greatly accelerated.

## Theory and methods

### A. TV-based photoacoustic reconstruction model

The algorithm proposed in this paper mainly targets two-dimensional computed-tomographic PAI for simple study. The possibility of extending the method to 3D will be discussed in “Discussion and conclusion”. In this imaging mode, laser pulses irradiate perpendicular to the image plane. Assuming that the tissue is irradiated uniformly by the laser, the relationship between the photoacoustic signals and the photoacoustic image can be described by the photoacoustic Equation [1]:

1 $$\nabla^{2} p({\mathbf{r}},t) - \frac{1}{{c^{2} }}\frac{{\partial^{2} p({\mathbf{r}},t)}}{{\partial t^{2} }} = - \frac{\beta }{{C_{p} }}A({\mathbf{r}}) \cdot \frac{\partial I(t)}{\partial t},$$

where *p*(**r**, *t*) is the photoacoustic signals at time *t* and position **r**, *c* is the speed of sound, *μ* is the isobaric expansion coefficient, *C*_*p*_ is the specific heat, *I*(*t*) is the temporal profile of the laser pulse and *A*(**r**) is the light absorption distribution of the tissue.

Assuming *I*(*t*) is an impulse signal and the sound velocity and other parameters of tissue are homogeneous, Eq. (1) can be solved by Green’s function [1]:

2 $$p({\mathbf{r}}_{0} ,t) = \frac{\beta }{{4\pi C_{p} }}\frac{\partial }{\partial t}\mathop{{\int\!\!\!\!\!\int}\mkern-21mu \bigcirc}\nolimits_{{\left| {{\mathbf{r}} - {\mathbf{r}}_{0} } \right| = ct}} {\frac{{A({\mathbf{r}})}}{t}d^{2} {\mathbf{r}}} ,$$

where **r**_**0**_ is the position of the ultrasound transducer.

Now, we establish the forward model from photoacoustic signals to a photoacoustic image. From Eq. (2), it can be derived that:

3 $$\frac{{4\pi C_{p} t}}{\beta }\int_{0}^{t} {p({\mathbf{r}}_{0} ,t)} dt = \mathop{{\int\!\!\!\!\!\int}\mkern-21mu \bigcirc}\nolimits_{{\left| {{\mathbf{r}} - {\mathbf{r}}_{0} } \right| = ct}} {A({\mathbf{r}})d^{2} {\mathbf{r}}} .$$

Define the product of detected photoacoustic signals at sampling points **r**_**0**_ and sampling time *t*, *g*(**r**_0_,*t*), as:

4 $$g({\mathbf{r}}_{0} ,t) = \frac{{4\pi C_{p} t}}{\beta }\int_{0}^{t} {p({\mathbf{r}}_{0} ,t)} dt,$$

Equation (3) can be rewritten as:

5 $$g({\mathbf{r}}_{0} ,t) = \mathop{{\int\!\!\!\!\!\int}\mkern-21mu \bigcirc}\nolimits_{{\left| {{\mathbf{r}}_{0} - {\mathbf{r}}} \right| = ct}} {A({\mathbf{r}})} d^{2} {\mathbf{r}}.$$

In practical applications, the images and sampling signals tend to be discretized and can be written in the form of a vector [34]:

6 $$\begin{array}{*{20}c} {{\mathbf{g}}_{l} = {\mathbf{M}}_{l}^{{\mathbf{T}}} \cdot {\mathbf{A}}',} \, {l = 1,2,3, \ldots,N,} \\ \end{array}$$

where **A** is the matrix of the photoacoustic image of size *N*_*x*_× *N*_*y*_, **A**′ is a column vector transposing **A**, *l* is the number of sampling points and **M**_*l*_ is weight matrix for the *l*th sampling point, **g**_*l*_ is the column vector discretized from *g*(**r**_0_, *t*) for the *l*th sampling point.

An image’s gray values tend to have no sparsity, while its discrete gradients have more sparsity under some circumstances, such as homogeneous distribution of light in the sample and piecewise constant absorption coefficient.

TV can be expressed as the *l*_1_ norm of the discrete gradient matrix of the image [62]:

7 $$TV({\mathbf{\rm A}}) = \sum\limits_{m,n} {\left[ {\left( {A_{m,n} - A_{m - 1,n} } \right)^{2} + \left( {A_{m,n} - A_{m,n - 1} } \right)^{2} } \right]^{1/2} } ,$$

where *A*_*m,n*_ is the gray value of the pixel at the position (*m*, *n*).

The optimization problem of TV-based photoacoustic reconstruction can be written as:

8 $${\mathbf{A}}^{ * } = { \text{arg} }\,\mathop { \text{min} }\limits_{{\mathbf{A}}} \left\| {{\mathbf{M}}^{{\mathbf{T}}} \cdot {\mathbf{A^{\prime}}} - {\mathbf{g}}} \right\|_{2}^{2} + \alpha TV({\mathbf{A}}),$$

where α is the parameter corresponding to the weight of TV value in the optimization. Equation (8) can also be written as:

9 $${\mathbf{A}}^{ * } = { \text{arg} }\,\mathop { \text{min} }\limits_{{\mathbf{A}}} \left\| {{\mathbf{M}}^{{\mathbf{T}}} \cdot {\mathbf{A}}^{'} - {\mathbf{g}}} \right\|_{2}^{2} + \alpha \sum\limits_{k} {\left| {{\mathbf{u}}_{k} } \right|_{2} } ,$$

where **u**_*i*_= *D*_*i*_**A**. *D*_*i*_ is a defined matrix that calculates the finite difference of **A** at the *i*th pixel.

### B. Nonlocal patch regular constraint

There can be many similar patches in an image. In the flat region, most pixels and patches are identical, while the texture and edge regions also show similarities. Buades et al. therefore proposed the nonlocal idea and extended the similarities between pixels to that between patches [53]. For the nonlocal idea, a neighborhood is no longer for pixels in the common sense but is rather the patch-set under a certain measure of similarity.

For pixel **x***i *= (*xi*_1_, *xi*_2_), *P*_**x***i*_ refers to the patch centered at **x***i*. The self-similarity of the image can be represented in terms of the similarities between patches:

10 $$P_{{{\mathbf{x}}i}} = \sum\limits_{{{\mathbf{x}}j \in \delta ({\mathbf{x}}i)}} {W({\mathbf{x}}i,{\mathbf{x}}j)P_{{{\mathbf{x}}j}} } ,$$

where *W*(**x**_*i*_, **x**_*j*_) is the weight function between *P*_**x***i*_ and P_**x***j*_. It measures the similarity degree between the two patches and satisfies $\sum\nolimits_{{{\mathbf{x}}j \in \delta ({\mathbf{x}}i)}} {W({\mathbf{x}}i,{\mathbf{x}}j)} = 1$. *δ*(**x**_*i*_) refers to the neighborhood of *P*_**x***i*_:

11 $$\delta \left( {{\mathbf{x}}i} \right) = \left\{ {{\mathbf{x}}j|W({\mathbf{x}}i,{\mathbf{x}}j) > T} \right\},$$

where *T* is a threshold value to screen the similar patches. If the weight is greater than T, these two patches are considered similar. Otherwise, this patch does not belong to the neighborhood of patch *P*_**x***i*_. Equation (11) represents the collection of every pixel whose similarity to patch *P*_**x***i*_ is greater than *T*.

There are multiple expressions for the weight function *W*(**x**_*i*_, **x**_*j*_), and it is usually inversely proportional to the distance between **x**_*i*_ and **x**_*j*_. These weight functions failed to maintain the structure and directivity information of the image. So they are not qualified for the adaptive selection of the neighborhood of the patches. Liu et al. proposed the direction adaptive weight function [59], which is adopted in this paper:

12 $$W_{s} ({\mathbf{x}}_{i} ,{\mathbf{x}}_{j} ) = \frac{{\sqrt {{ \det }(S_{j} )} }}{{2\pi h^{2} \mu_{j}^{2} }}\exp \left\{ { - \frac{{({\mathbf{x}}_{i} - {\mathbf{x}}_{j} )^{{\mathbf{T}}} S_{j} ({\mathbf{x}}_{i} - {\mathbf{x}}_{j} )}}{{2h^{2} \mu_{j}^{2} }}} \right\},$$

where *S*_*j*_ is the modified structure tensor matrix. *h* is the global smoothing parameter and *μ*_*i*_ is the local density of samples data. More details can be found in Ref. [59]. The structure tensor matrix *S*_*j*_ reflects the information of gray values and gradients for the image. Using this direction-adaptive weight function, the neighborhood *δ*(**x**_*i*_) of patch *P*_**x***i*_ can be adaptively selected. The selection of the neighborhood takes the directivity and geometric structure of the image fully into consideration, so it can provide more reliable estimations for the weight calculation between patches. Therefore, the structure and directivity information of the image can be well maintained.

The nonlocal patch regular constraint corresponding to the self-similarity between patches in Eq. (2) can be written as:

13 $${ \text{min} }\left( {\sum\limits_{i} {\left( {P_{{{\mathbf{x}}i}} - \sum\limits_{{{\mathbf{x}}j \in \delta ({\mathbf{x}}i)}} {W_{s} ({\mathbf{x}}i,{\mathbf{x}}j)P_{{{\mathbf{x}}j}} } } \right)^{2} } } \right).$$

Patch *P*_x*i*_ is estimated by using the weights of patches in the neighborhood that have the highest similarities to *P*_x*i*_. It is the first time that nonlocal-patch is applied as the regularized constraint for the reconstruction of image in PAI. By the constraint of the nonlocal patch, the problem concerning the inaccuracy of the similarity estimation through the use of isolated pixel points is surmounted, and the structure information, such as edges and texture, can be well preserved.

### C. Patch-TV photoacoustic reconstruction algorithm

The TV-based reconstruction model in Eq. (9) has good performance, but it fails to preserve the geometric structure of the image. To solve the problems of TV and make reconstruction algorithms more suitable for practical application, the nonlocal patch regular constraint is incorporated into the TV-based regular term:

14 $${\mathbf{A}}^{ * } = { \text{arg} }\,\mathop { \text{min} }\limits_{{\mathbf{A}}} \left( {\left\| {{\mathbf{M}}^{{\mathbf{T}}} \cdot {\mathbf{A^{\prime}}} - g} \right\|_{2}^{2} + \alpha \sum\limits_{i} {\left| {u_{i} } \right|_{2} + \beta } \sum\limits_{i} {\left\| {\left( {P_{{{\mathbf{x}}i}} - \sum\limits_{{{\mathbf{x}}j \in \delta ({\mathbf{x}}i)}} {W_{s} ({\mathbf{x}}i,{\mathbf{x}}j)P_{{{\mathbf{x}}j}} } } \right)} \right\|_{2}^{2} } } \right).$$

where *β* is the parameter corresponding to the weight of local-patch value in the optimization. Define the nonlocal matrix **H** consisting of the weight functions *W*_*s*_(**x**_*i*_, **x**_*j*_) [63]:

15 $${\mathbf{H}} = (a_{ij} )_{{N^{2} \times M^{2} }} \, a_{ij} = \left\{ {\begin{array}{*{20}l} {W_{s} ({\mathbf{x}}i,{\mathbf{x}}j),\quad {\mathbf{x}}j \in \delta ({\mathbf{x}}i) \, } \\ {0,\quad \quad \quad \quad {\mathbf{x}}j \notin \delta ({\mathbf{x}}i) \, } \\ \end{array} } \right\}.$$

When **x**_*j*_ is in the neighborhood *δ*(**x**_*i*_) of **x**_*i*_, *α*_*ij*_ in **H** is set to the weight *W*_*s*_(**x**_*i*_, **x**_*j*_). When **x**_*j*_ is not in the neighborhood *δ*(**x**_*i*_) of **x**_*i*_, *α*_*ij*_ is set to 0. In this way the summation item in the constraint item of local-patch can be expressed as multiplication between matrix **H** and **A**. Define **H**′ expressing the transversal vector transposing **H**. The size of **H**′ is 1 × (N^2^ × M^2^). The optimization problem in Eq. (14) can be rewritten into the form of a matrix:

16 $${\mathbf{A}}^{ * } = { \text{arg} }\,\mathop { \text{min} }\limits_{{\mathbf{A}}} \left( {\left\| {{\mathbf{M}}^{{\mathbf{T}}} \cdot {\mathbf{A^{\prime}}} - {\mathbf{g}}} \right\|_{2}^{2} + \alpha \sum\limits_{i} {\left| {{\mathbf{u}}_{i} } \right|_{2} + \beta } \sum\limits_{i} {\left\| {\left( {{\rm I}^{\prime} - {\mathbf{H^{\prime}}}} \right){\mathbf{{\rm A}^{\prime}}}} \right\|_{2}^{2} } } \right),$$

where **I**′ with the same size with that of **H**′ is the transversal vector transposing the unit matrix **I**. Combine the first and third terms in Eq. (16) in matrix form:

17 $${\mathbf{A}}^{ * } = { \text{arg} }\,\mathop { \text{min} }\limits_{{\mathbf{A}}} \left( {\left\| {\left[ {\begin{array}{*{20}c} {\mathbf{g}} \\ 0 \\ \end{array} } \right] - \left[ {\begin{array}{*{20}c} {{\mathbf{M}}^{{\mathbf{T}}} } \\ {\beta ({\mathbf{{\rm I}^{\prime}}} - {\mathbf{H^{\prime}}})} \\ \end{array} } \right]{\mathbf{A^{\prime}}}} \right\| + \alpha \sum\limits_{i} {\left| {{\mathbf{u}}_{i} } \right|_{2} } } \right).$$

Using the notation $${\tilde{\mathbf{g}}} = \left[ {\begin{array}{*{20}c} {\mathbf{g}} \\ 0 \\ \end{array} } \right], \, {\mathbf{K}} = \left[ {\begin{array}{*{20}c} {{\mathbf{M}}^{{\mathbf{T}}} } \\ {\beta ({\mathbf{\rm I}}^{'} - {\mathbf{H}}^{'} )} \\ \end{array} } \right],$$ Eq. (17) can be simplified as:

18 $$\begin{aligned} & {\mathbf{A}}^{ * } = {\text{arg} }\, \mathop{ \text{min} }\limits_{{\mathbf{A}}} \left( {\left\| {{\mathbf{\rm K}} \cdot {\mathbf{A^{\prime}}} - {\tilde{\mathbf{g}}}} \right\|_{2}^{2} + \alpha \sum\limits_{i} {\left| {{\mathbf{u}}_{i} } \right|_{2} } } \right) \\ & \quad \quad \quad \quad \quad s.t. \, {\mathbf{u}}_{i} = D_{i} {\mathbf{A}}. \\ \end{aligned}$$

The patch-TV optimization problem is simplified to a common photoacoustic iterative reconstruction model. The variable splitting and Barzilai–Borwein-based method is employed to solve the optimization problem in Eq. (18) [60]. This method has excellent performance in rapidly solving photoacoustic reconstruction regularized problems. Using the standard augmented Lagrangian method and the Barzilai–Borwein step size to accelerate the convergence speed, Eq. (19) can be deduced as [60, 64]:

19 $$\begin{aligned} ({\mathbf{u}}^{n + 1} ,{\mathbf{A}}^{{{\text{n}} + 1}} ) &= { \text{min} }_{{{\mathbf{u,A}}}} \left\{ {\alpha \sum\limits_{i} {\left( {\left| {{\mathbf{u}}_{i} } \right|_{2} + \left| {{\mathbf{u}}_{i} - D_{i} {\mathbf{A}}^{n} - b_{i}^{n} } \right|_{2}^{2} } \right)} } \right. \\ & \quad \left. { +\, \sigma_{n} \left( {\left| {{\mathbf{u}}^{{{\text{n}} + 1}} - {\mathbf{u}}^{\text{n}} } \right|_{2}^{2} + \frac{1}{2}\left| {{\mathbf{u}} - {\mathbf{u}}^{n} + \sigma_{n}^{ - 1} {\mathbf{K}}^{\text{T}} ({\mathbf{K}}^{T} {\mathbf{A}}^{n} - \tilde{g} )} \right|_{2}^{2} } \right)} \right\}, \hfill \\ \end{aligned}$$

where *b*_*k*_^*n*^ is the TV step parameter in the *n*th iteration and *σ*_*n*_ is the defined Barzilai–Borwein step size in the *n*th iteration. By using the variable splitting method, Eq. (20) can be translated into the following two sub-problems:

20 $$\begin{aligned} & {\mathbf{u}}_{i}^{n + 1} = { \text{min} }_{{u_{i} }} \left\{ {\left| {{\mathbf{u}}_{i} } \right|_{2} + \left| {{\mathbf{u}}_{i} - D_{i} {\rm A}^{n} - b_{i}^{n} } \right|_{2}^{2} + \frac{{\delta_{i} }}{\alpha }\left| {{\mathbf{u}}_{i} - {\mathbf{u}}_{i}^{n} } \right|_{2}^{2} } \right\}, \\ & {\mathbf{A}}^{{{\text{n}} + 1}} = { \text{min} }_{\text{A}} \left\{ {\alpha \left| {D{\rm A} - {\mathbf{u}}^{{{\text{n}} + 1}} } \right|_{2}^{2} + \delta_{n} \left| {{\mathbf{A}} - \left( {{\mathbf{A}}^{n} - \sigma_{n}^{ - 1} {\mathbf{K}}^{\text{T}} ({\mathbf{KA}}^{n} - {\mathbf{g}} )} \right)} \right|_{2}^{2} } \right\} \\ & b_{i}^{n + 1} = b_{i}^{n} - \left( {{\mathbf{u}}_{i}^{n + 1} - D_{i} {\mathbf{A}}^{n + 1} } \right), \\ & \sigma_{n + 1} = \left( {{{\left| {{\mathbf{K}}\left( {{\mathbf{A}}^{{{\text{n}} + 1}} - {\mathbf{A}}^{\text{n}} } \right)} \right|_{2}^{2} } \mathord{\left/ {\vphantom {{\left| {{\mathbf{K}}\left( {{\mathbf{A}}^{{{\text{n}} + 1}} - {\mathbf{A}}^{\text{n}} } \right)} \right|_{2}^{2} } {\left( {\left| {{\mathbf{u}}^{{{\text{n}} + 1}} - {\mathbf{u}}^{\text{n}} } \right|_{2}^{2} + \left| {{\mathbf{A}}^{{{\text{n}} + 1}} - {\mathbf{A}}^{\text{n}} } \right|_{2}^{2} } \right)}}} \right. \kern-0pt} {\left( {\left| {{\mathbf{u}}^{{{\text{n}} + 1}} - {\mathbf{u}}^{\text{n}} } \right|_{2}^{2} + \left| {{\mathbf{A}}^{{{\text{n}} + 1}} - {\mathbf{A}}^{\text{n}} } \right|_{2}^{2} } \right)}}} \right.. \\ \end{aligned}$$

The two sub-problems can be solved using the shrinkage operator method [60]:

21 $$\left\{ {\begin{array}{*{20}l} {{\mathbf{u}}_{i}^{n + 1} = { \text{max} }\left\{ {\left\| {\frac{{a_{1} + a_{2} \delta_{n} /\alpha }}{{a_{1} + a_{2} }}} \right\| - \frac{1}{{a_{1} + a_{2} }},0} \right\}\frac{{1/(a_{1} + a_{2} )}}{{\left\| {1/(a_{1} + a_{2} )} \right\|}}} \\ {a_{1} = D_{i} {\mathbf{A}}^{n} + b_{i}^{n} \quad \quad \quad \quad \quad \quad \quad \quad \quad \quad \quad (i = 1,2 \ldots N_{x} N_{y} ),} \\ {a_{2} = {\mathbf{u}}_{i}^{n} } \\ \end{array} } \right.$$

22 $${\mathbf{A}}^{n + 1} = F^{\text{T}} \left\{ {\frac{{F[\alpha \,D^{\text{T}} {\mathbf{u}}^{n + 1} + \sigma_{n} {\mathbf{A}}^{n} - {\mathbf{K}}^{T} ({\mathbf{KA}}^{n} - {\tilde{\mathbf{g}}})]}}{{\alpha \,F^{\text{T}} D^{\text{T}} DF + \sigma_{n} {\mathbf{I}}}}} \right\}.$$

where *F* is the Fourier transform matrix.

The flow of the patch-TV photoacoustic reconstruction algorithm can be summarized as follows:

1. Initialization: Input **A**, *α, β*, *T*. Set the reconstructed image **A**^0^ = **0**, *δ*_0_ = 1, and *b*^0^ = 0.
2. Apply Eq. (21) to update **u**^*n*^ for the given **A**^*n*−**1*′***^.
3. Apply Eq. (22) to update **A**^*n*^ for the given **u**^*n*^.
4. Apply Eq. (22) to update *b*^*n*^ and *δ*_*n*_.
5. If the terminal condition is met, end the iteration. Otherwise, let *n* = *n *+ 1, and return to steps 2–4. The termination condition is as follows:
  23 $$\frac{{\left\| {u^{n} - u^{n - 1} } \right\|}}{{\left\| {u^{n} } \right\|}} < \varepsilon .$$

## Numerical simulation

To verify the reconstruction quality and performance of the proposed patch-TV algorithm, a variety of numerical simulations are designed and conducted. To simulate the signal collection in practice, straight-line scanning with varying sampling points is executed. Straight-line scanning in different directions to the phantom is also tested to validate the universality of the algorithm. The Shepp–Logan phantom, which is widely used in biomedical imaging, and the FORBILD phantom [65], which is more complicated and challenging, are chosen in the simulations. The results for the patch-TV algorithm are compared to those of the TV-GD and TV-Lp algorithms. The PSNR, the noise robustness and the convergence of the algorithms are also compared and discussed. The simulations are carried out using Matlab R2013a on a personal computer with a 2.4 GHz Intel(R) Xeon^®^ CPU and 64 GB memory. In the simulations, the sampling frequency is 200 MHz and the recording time of pressure waves is 20 μs for all the cases. The simulations for the signals and reconstructions are all conducted in the same two-dimensional plane.

### A. Straight-line scanning

**Fig. 1:**
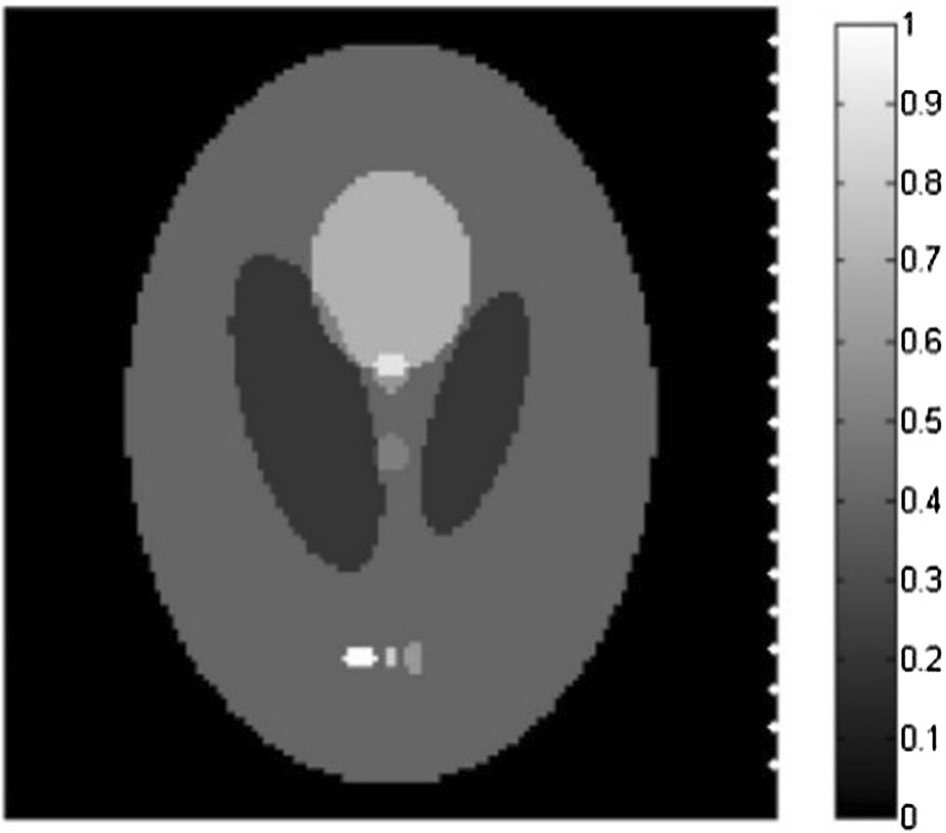
The Shepp–Logan phantom and a diagram of the straight-line scanning in the perpendicular direction

First, the Shepp–Logan phantom is adopted as the initial pressure rise distribution, which is shown in Fig. 1. The size of the phantom is 76.8 × 76.8 mm, and the reconstructed images size is set to 128 × 128 pixels. The scanning line on the right side of the phantom with the length of 76 mm is also shown in Fig. 1, from which we can see that the scanning line is parallel to the major axis of the ellipse of the phantom. We use the photoacoustic equation (Eq. 3 in paper) for the numerically produced simulated data and the forward projection model we described in the paper to reconstructed the image iteratively under patch-TV regulation. Thus the inverse crime is avoided in our method during the generation of simulated signals. The distance from the center of the image to the scanning line is 38 mm. The length of the scanning line remains constant, while the sampling points can be 10, 20, or 50. The iteration number is set to 10 for all algorithms. The parameter settings for patch-TV are estimated by testing the values that provides the best performance for the simulations. In this case, *α* = 0.4, *β *= 0.35, *T *= 0.65. The parameters for TV-GD and TV-Lp are set referring [34, 39] to achieve the best performance in the simulations. The parameter settings for these algorithms are also estimated by testing the values that provides the best performance for the simulations.

**Fig. 2:**
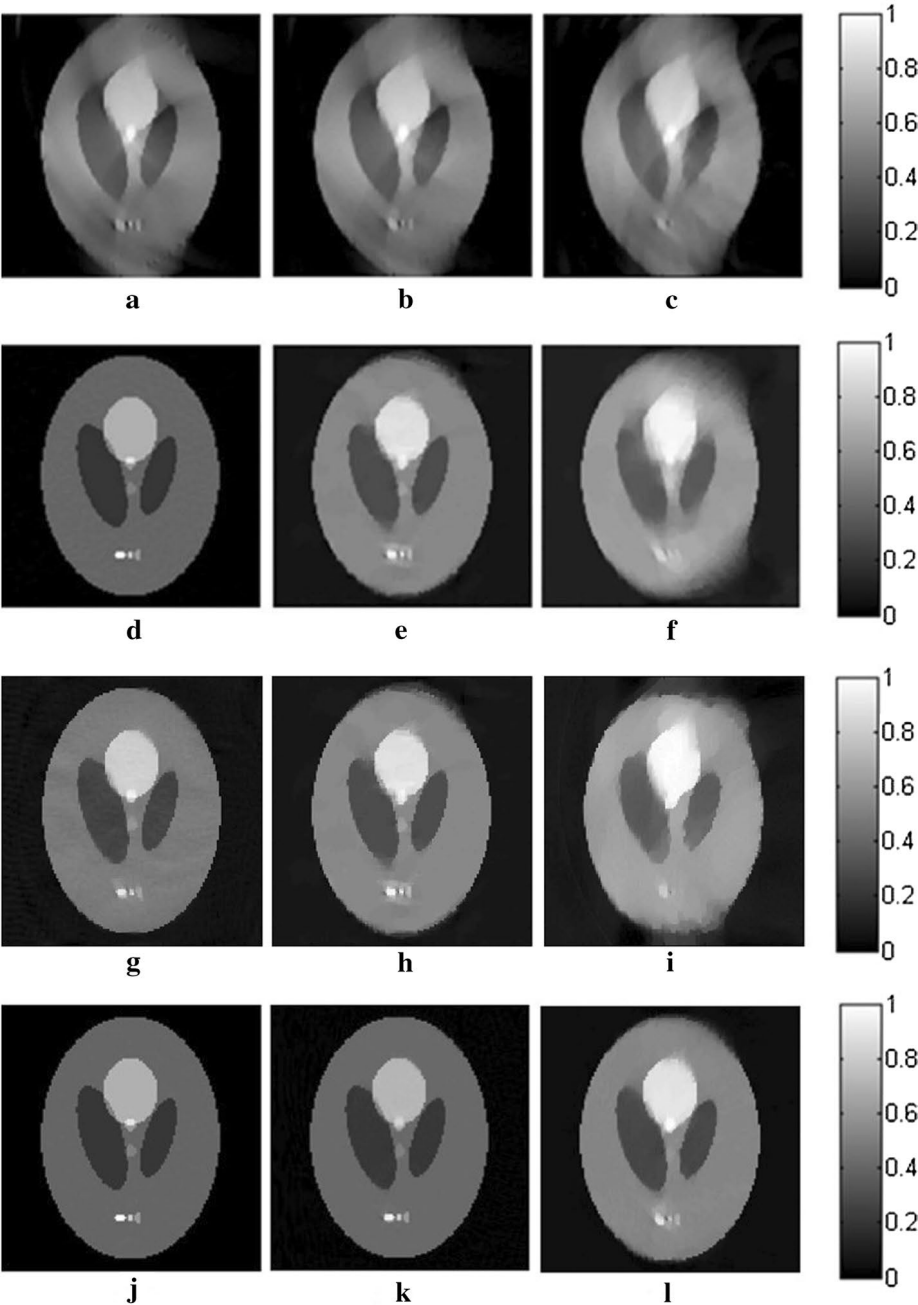
The reconstructed results for straight-line scanning of the Shepp–Logan phantom in the perpendicular direction for TV-GD (**a**–**c**), TV-Lp (**d**–**f**), Patch-RE (**g**–**i**) and patch-TV (**j**–**l**). The first, second, and third columns refer to the results for 50- (**a**, **d**, **g**, **j**), 20- (**b**, **e**, **h**, **k**), and 10-point (**c**, **f**, **i**, **l**) sampling, respectively

The reconstruction results for the three algorithms are shown in Fig. 2. The images in this paper are normalized in the same gray level for comparison. The gray values of all pixels are divided by the maximum one in the images to avoid any effect to the quality of the images. In the first row of Fig. 2, the reconstructed images for TV-GD have serious artifacts and blurred edges, which severely distort the images, especially in the vertical direction, where the angular information is missing. Regarding TV-Lp in the second row of Fig. 2, the result is improved over that of TV-GD when the sampling points are sufficient. However, the quality of the reconstruction declines rapidly as the number of the sampling points decreases. We can see that for the 10-point sparse-view reconstruction in Fig. 2f, there is serious vagueness in the perpendicular direction of the image. As for Patch-RE, in the third line, the results are even worse than those of TV-Lp and just slightly better than those of TV-GD. It is because without TV-optimization to ensure the quality of the image in each iteration, the effects of the patch regularization will be greatly weaken. The results of patch-TV in the third row of Fig. 2 show great improvement over the other two algorithms. The artifacts are effectively suppressed, and the edges of the image are distinct. The geometric structure of the images is preserved well, with almost no blur or distortion. Furthermore, a sharp decrease in the number of sampling points does not have a great effect on the quality of the reconstructed image.

The PSNRs of the reconstruction results for the four algorithms are also calculated and compared as the quantitative criteria for the evaluation of the reconstruction results. The greater the value of PSNR is, the better the reconstruction. The calculation formula of the PSNR is as follows:

24 $$PSNR = 10 \cdot \log_{10} \left( {\frac{{N_{x} N_{y} \cdot MAXI^{2} }}{{\sum\nolimits_{m = 1}^{{N_{x} }} {\sum\nolimits_{n = 1}^{{N_{y} }} {\left( {A_{m,n} - R_{m,n} } \right)^{2} } } }}} \right),$$

where *R*_*m,n*_ is the gray value of the original image and MAXI is the maximum possible pixel value of the image. The original images which were not normalized are used for all the PSNR calculations in this paper. The PSNR results are displayed in Table 1.

**Table 1:** PSNRs (dB) of the straight-line scanning of the Shepp–Logan phantom in the vertical direction

PSNRs (dB)	50 points	20 points	10 points
TV-GD	17.58	16.46	14.35
TV-Lp	26.97	20.54	15.39
Patch-RE	21.47	18.03	14.29
Patch-TV	34.98	31.35	23.49

Table 1 shows that patch-TV obtains the highest PSNR values for every case. The PSNR values for TV-GD are always low on account of the deficiency of the data for straight-line scanning. In fact, the results of TV-GD, are poor in all kinds of sampling condition even though when the sampling points are sufficient (50-points). We can see that the PSNRs of TV-GD are all lower than 20 dB. Under this circumstance, the amount of variation of PSNRs actually does not make much sense. TV-Lp has a good PSNR for 50-point scanning, but the value of the PSNR decreases rapidly as the number of sampling points decreases. The PSNRSs of Patch-RE are just slightly higher than that of TV-GD. On average, the PSNR of patch-TV is approximately 17 dB higher than that of TV-GD, 8 dB higher than that of TV-Lp and 12 dB higher than that of Patch-RE.

**Fig. 3:**
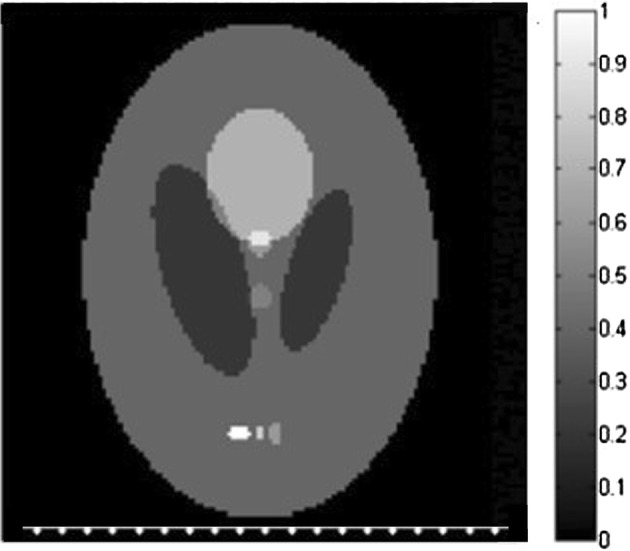
The Shepp–Logan phantom and the diagram of the straight-line scanning in the horizontal direction

To test the universality of the algorithm in practical applications, we change the position of the scanning line relative to the phantom. In this case, the scanning line is parallel to the minor axis of the ellipse of the image. Its length and the distance to the center of the image remain unchanged. The numbers of sampling points are again 50, 20 and 10. The diagram of the scanning line is shown in Fig. 3. The parameter settings in this case is *α* = 0.50, *β *= 0.42, *T *= 0.65.

**Fig. 4:**
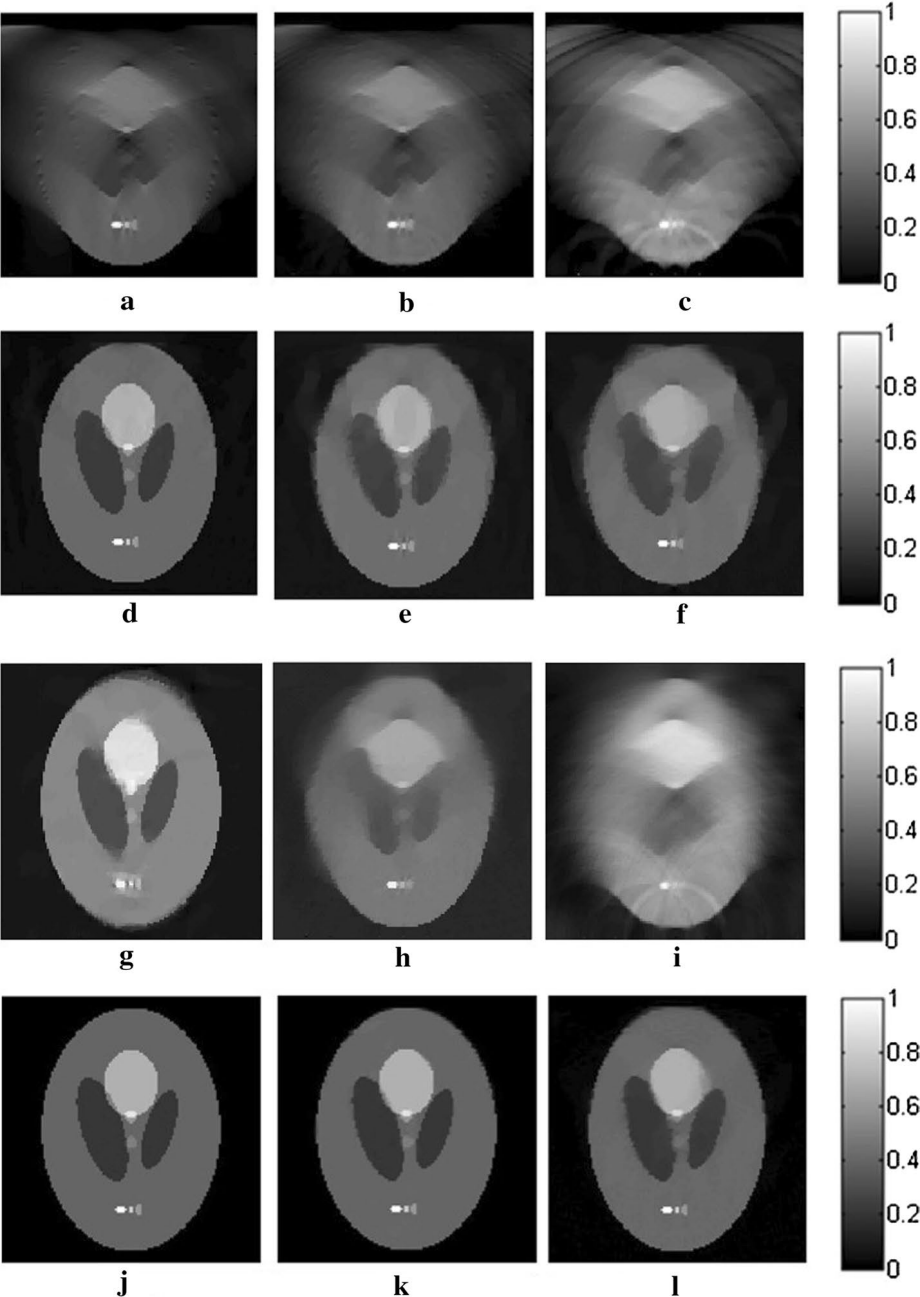
The reconstructed results for straight-line scanning of the Shepp–Logan phantom in the horizontal direction for TV-GD (**a**–**c**), TV-Lp (**d**–**f**), Patch-RE (**g**–**i**) and patch-TV (**j**–**l**). The first, second, and third columns refer to the results for 50- (**a**, **d**, **g**, **j**), 20- (**b**, **e**, **h**, **k**), and 10-point (**c**, **f**, **i**, **l**) sampling, respectively

The results of the reconstruction for the three algorithms are shown in Fig. 4. We can see that there are a large number of blurs and distortions in the reconstructed images for TV-GD, especially in the horizontal direction. The geometry structure information of the image is destroyed. TV-Lp and Patch-RE fails to obtain ideal results, especially when the sampling points become sparse. Regarding patch-TV, the edges and texture structure of the image are better preserved. The artifacts and background noise are effectively suppressed. Even in sparse-view scanning, there is almost no blurring in the image.

We also compare the PSNRs of the results for the three algorithms in Table 2. The PSNR of patch-TV is approximately 18 dB higher than that of TV-GD, 10 dB higher than that of TV-Lp, on average and 14 dB higher than that of Patch-RE.

**Table 2:** PSNRs (dB) of the straight-line scanning of the Shepp–Logan phantom in the horizontal direction

PSNRs (dB)	50 points	20 points	10 points
TV-GD	17.74	15.87	13.48
TV-Lp	25.29	19.28	15.97
Patch-RE	20.43	16.34	13.69
Patch-TV	34.68	32.13	23.70

**Fig. 5:**
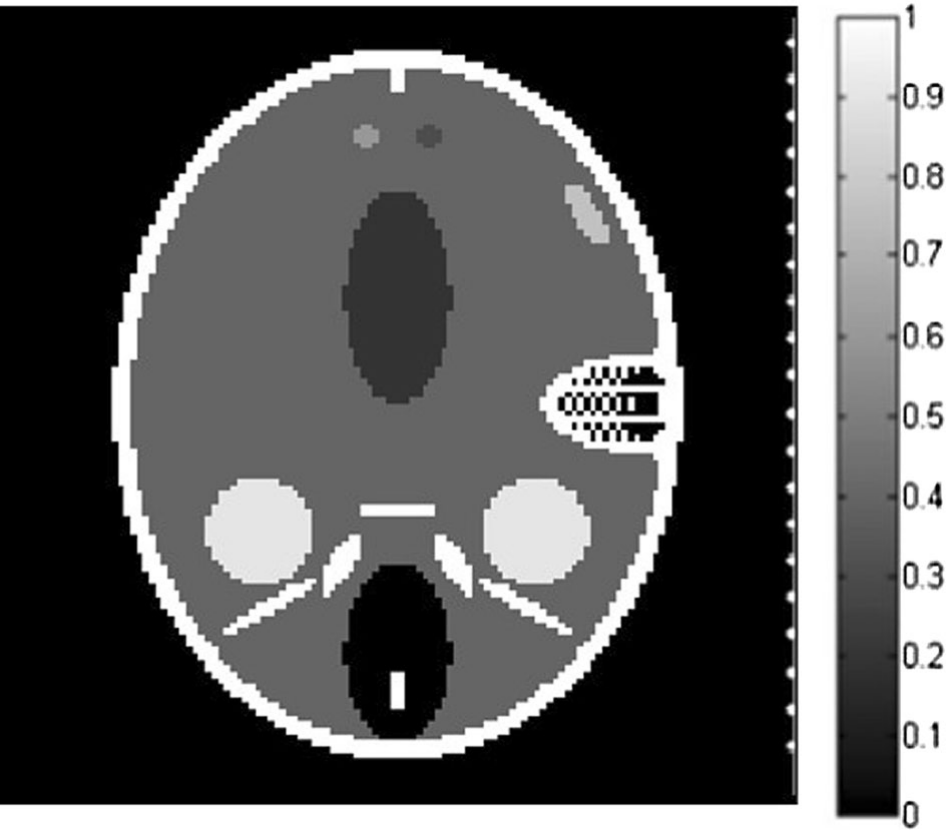
The FORBILD phantom and the diagram of the straight-line scanning in the perpendicular direction

**Fig. 6:**
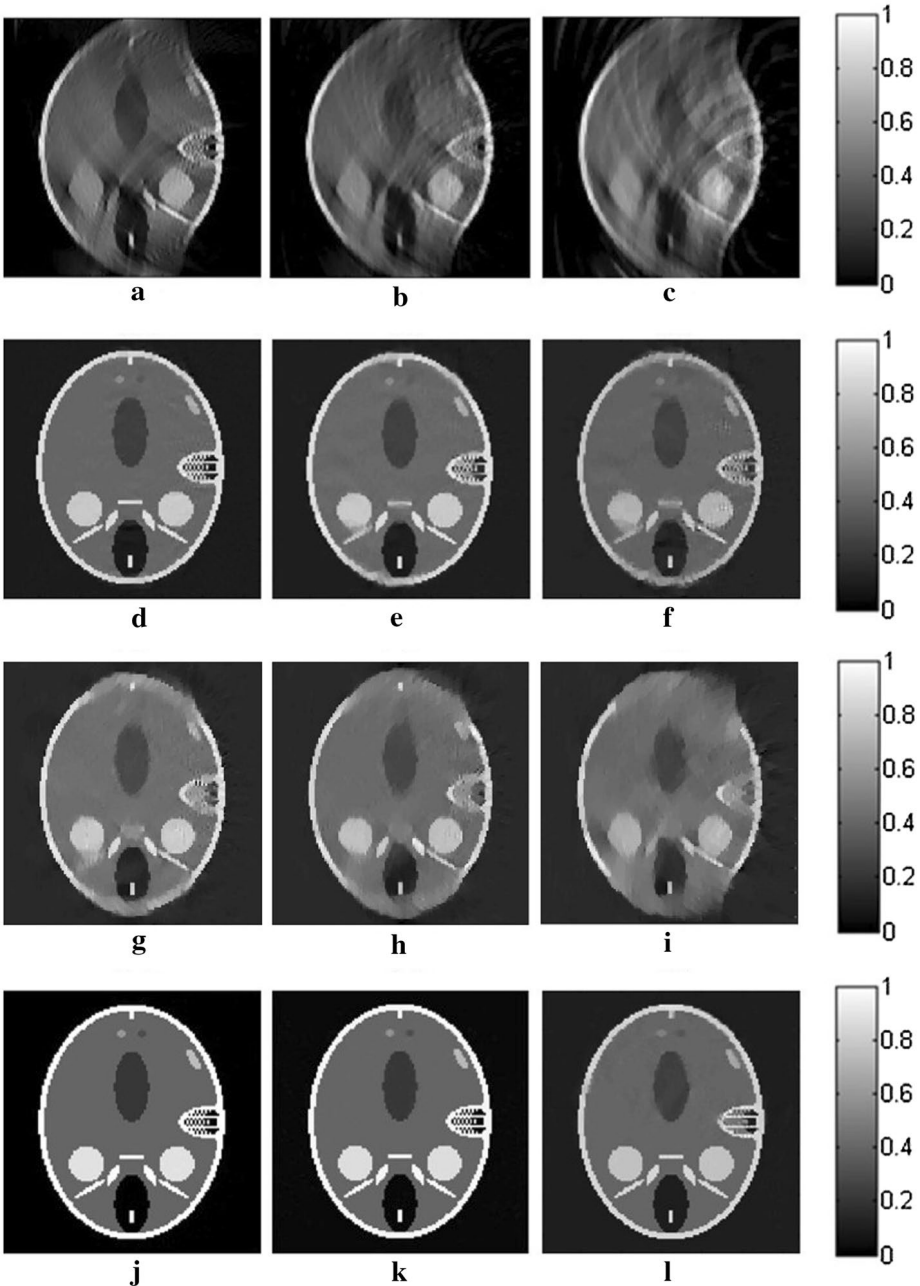
The reconstructed results for straight-line scanning of the FORBILD phantom for TV-GD (**a**–**c**), TV-Lp (**d**–**f**), Patch-RE (**g**–**i**) and patch-TV (**j**–**l**). The first, second, and third columns refer to the results for 50- (**a**, **d**, **g**, **j**), 20- (**b**, **e**, **h**, **k**), and 10-point (**c**, **f**, **i**, **l**) sampling, respectively

To further validate the effectiveness of the proposed algorithm, the FORBILD phantom, which is more complex and challenging, is also adopted in the simulation. The phantom and the scanning line are shown in Fig. 5. The size of the phantom and the scanning settings are the same as those in Fig. 1. Fifty-, 20-, and 10-point straight-line reconstructions are conducted, and the results of the three algorithms are shown in Fig. 6. The parameter settings in this case is *α* = 0.65, *β *= 0.54, *T *= 0.57. TV-GD and Patch-RE shows poor performance, yielding bad image quality. The incompleteness of the data has a significant effect on the reconstruction. For TV-Lp, serious artifacts and blurring occur when the number of sampling points decreases. The contrasts of the images are not high, and the performance is not satisfactory. Patch-TV overcomes these problems. The geometric structure of the phantom is distinct, and the artifacts are effectively suppressed.

The PSNR results of the three algorithms are displayed in Table 3. It is obvious that patch-TV outperforms the other three algorithms for each sampling status, making the patch-TV algorithm superior to the other two algorithms even for a complicated phantom.

**Table 3:** PSNRs (dB) of the straight-line scanning of the FORBILD phantom

PSNRs (dB)	50 points	20 points	10 points
TV-GD	15.78	15.35	13.23
TV-Lp	22.43	16.87	14.37
Patch-RE	19.34	16.01	13.27
Patch-TV	31.54	26.14	22.23

### B. Noise robustness

In PAI practical applications, it is important that the reconstruction algorithms have excellent noise robustness because the detected photoacoustic signals are usually disturbed by the system noise. The system noise follows a Gaussian distribution. To test the noise robustness of the proposed algorithm, the 20-point sampled signals for the FORBILD phantom in “Straight-line scanning” are supplemented with white noise and a signal-to-noise ratio (SNR) of 10 dB, 5 dB or 0 dB. The parameter settings in this case is *α* = 0.73, *β *= 0.60, *T *= 0.54.

**Fig. 7:**
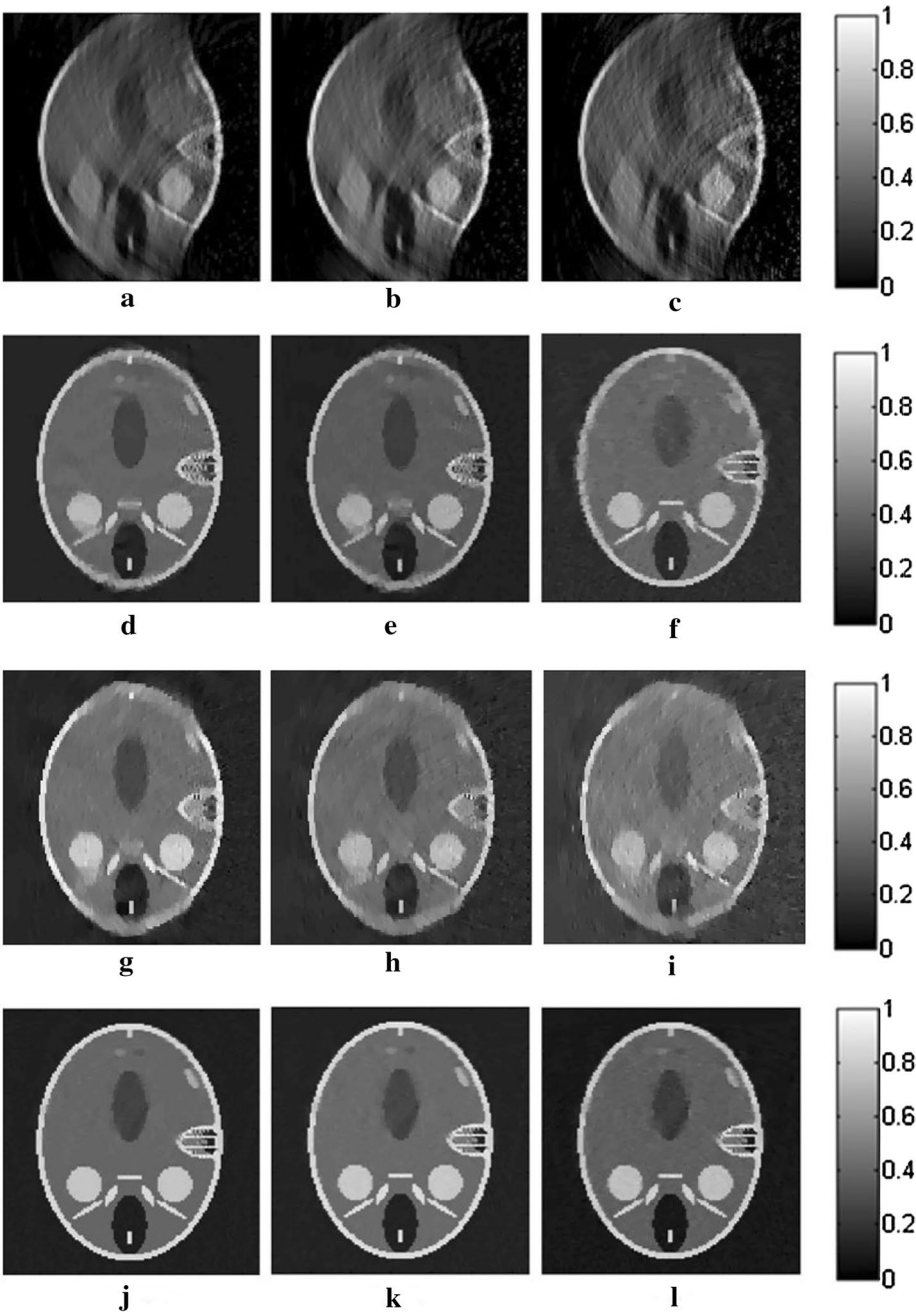
The images reconstructed from the noise-added signals by the TV-GD (**a**–**c**), TV-Lp (**d**–**f**), Patch-RE (**g**–**i**) and patch-TV (**j**–**l**). The first, second, and third columns refer to the results for a SNR of 10 dB (**a**, **d**, **g**, **j**), 5 dB (**b**, **e**, **h**, **k**), and 0 dB (**c**, **f**, **i**, **l**), respectively

The reconstructed results for the three algorithms for the different SNR signals are shown in Fig. 7. TV-GD, TV-Lp as well as Patch-RE fail to maintain high performance, especially at a low SNR. The quality of the images decays seriously, the contrasts of the images decrease, and the artifacts and background noise cannot be suppressed or eliminated. Patch-TV shows the highest performance in terms of noise robustness. The geometric structures of the reconstructed images are closer to those of the original image, and the noise is effectively suppressed.

The PSNRs of the reconstruction results are also displayed in Table 4. Patch-TV outperforms the other three algorithms, and the advantages are more obvious when the noise energy is stronger.

**Table 4:** PSNRs (dB) of noised signals for the FORBILD phantom

SNRs (dB)	10	5	0
TV-GD	13.46	12.98	11.18
TV-Lp	15.43	14.87	12.37
Patch-RE	14.46	13.23	11.36
Patch-TV	24.98	21.89	20.63

### C. Convergence and calculation

The convergence speed and calculation time are two other important performance indices for a photoacoustic iterative reconstruction algorithm. We define the distance between the reconstructed image and original image *d* as the quantization parameter:

25 $$d = \left( {\frac{{\sum\nolimits_{m = 1}^{{N_{x} }} {\sum\nolimits_{n = 1}^{{N_{y} }} {(A_{m,n} - R_{m,n} )^{2} } } }}{{\sum\nolimits_{m = 1}^{{N_{x} }} {\sum\nolimits_{n = 1}^{{N_{y} }} {R_{m,n}^{2} } } }}} \right)^{1/2} .$$

**Fig. 8:**
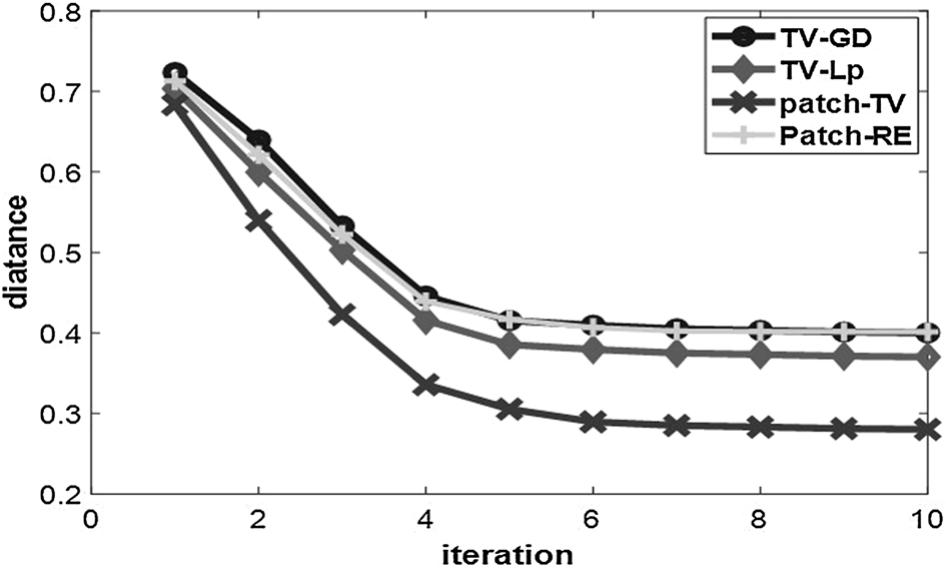
Line chart of the distance between the reconstructed image and the original image for each iteration of the TV-GD, TV-Lp, Patch-RE and patch-TV algorithms

The smaller *d* is, the smaller the difference between the reconstructed image and original image. We record *d* for every iteration step from 10-point sampling of the FORBILD phantom in “Straight-line scanning” and compare the *d* values of the four algorithms in each iteration in a line chart in Fig. 8. The results show that in every step, patch-TV’s *d* value is smaller than those of the other three algorithms, and it convergences to the smallest value.

The time costs *t* of 50-, 20-, and 10-point straight-line reconstruction of the Shepp–Logan phantom in “Straight-line scanning” for all four algorithms are also compared (Table 5). *t* calculates the time from input of the simulated data into the reconstruction algorithm to the output of the reconstructed image. The unit of *t* is second. The Barzilai–Borwe in method used in TV-Lp greatly accelerates the speed of the algorithm, and TV-Lp shows a greatly decreased time compared to TV-GD. For patch-TV, due to the incorporation of the nonlocal patch regularization, the time costs are higher than those of TV-GD, TV-Lp and Patch-RE. However, the performance of the algorithm is greatly improved, and the quality of the reconstructed images is enhanced significantly for practical applications.

**Table 5:** Calculation cost for the straight-line reconstruction of the Shepp–Logan phantom

*t* (s)	50 points	20 points	10 points
TV–GD	19.34	16.39	10.59
TV–Lp	14.69	10.48	6.39
Patch-RE	20.87	18.54	14.59
Patch-TV	24.58	20.38	17.23

According to the above simulations and discussion, patch-TV is superior to the two popular TV-based algorithms and is a highly efficient photoacoustic image reconstruction algorithm.

## Experimental results

To further validate and analyze the performance and practicability of the proposed algorithm, in vitro experiments were conducted. We used a single-detector platform to scan the Gelatin phantom linearly.

**Fig. 9:**
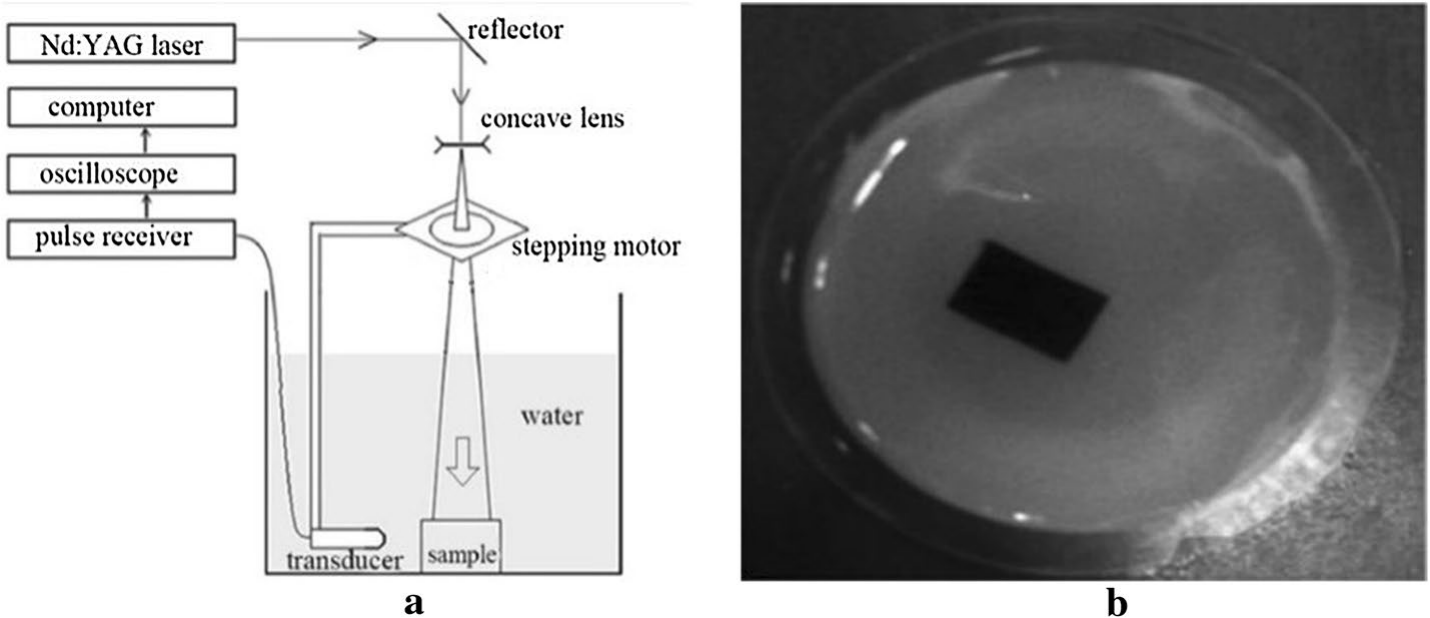
Scheme of the experimental platform for a single detector (**a**) and picture of the phantom used in the experiment (**b**)

The diagram of the single-detector platform is shown in Fig. 9a. It included a Nd:YAG laser device (Surelite I, Continuum, San Jose, California, USA) to emit a laser pulse with a wavelength of 532 nm and a frequency of 10 Hz. The duration of the laser pulse was 4–6 ns. A single transducer (V383-SU, Panametrics, Waltham, Massachusetts, USA) with a center frequency of 3.5 MHz and a bandwidth of 1.12 MHz was driven by a stepping motor scanning in the imaging plane. The sampling rate of the system was 16.67 MHz. The sampling frequency of the system is 16.67 MHz and the recording time of pressure waves is 50 μs. The experiment satisfied the American National Standards Institute (ANSI) laser radiation safety standard. The phantom for the straight-line scanning is shown in Fig. 9b. The phantom was made of a gelatin cylinder with a black rectangular rubber sheet embedded into it as a light absorber. The radius of the cylinder was 25 mm, and the size of the light absorber was 9 × 14 mm. The scanning line, which was parallel to the longer side of the light absorber, was uniformly distributed with 41 sampling points. The sampling interval was 1 mm. The perpendicular distance from the center of the phantom to the scan line was 45 mm. The radius of the phantom was 25 mm the reconstructed images size was also set to 128 × 128 pixels. The parameter settings in this case is *α* = 0.55, *β *= 0.45, *T* = 0.60.

**Fig. 10:**
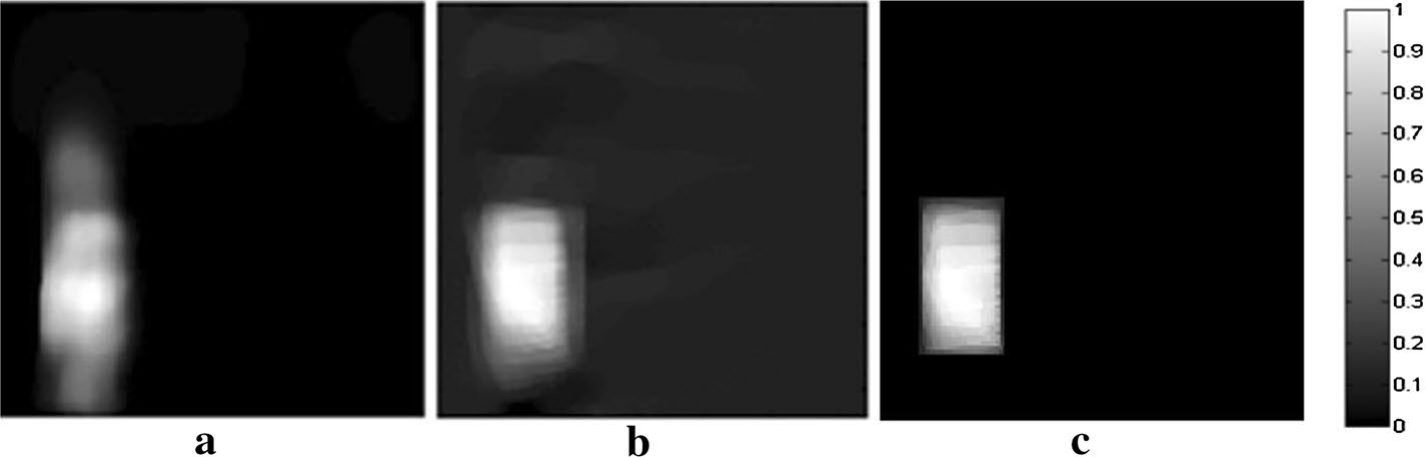
The reconstructed images of the phantom in Fig. 9b for the TV-GD (**a**), TV-Lp (**b**) and patch-TV (**c**) algorithms in the single-detector experiment

The reconstructed results for patch-TV, TV-Lp and TV-GD are shown in Fig. 10. Patch-TV obtained the best imaging quality. There were serious artifacts and blurring in the images for the other two algorithms. Particularly for TV-GD, serious distortions occurred in the vertical direction of the light absorber. The edges of the image were hard to recognize. The patch-TV result was greatly improved. The edges of the image were distinct, and the distribution of the gray values was relatively uniform. Furthermore, the artifacts and background noise were effectively suppressed. This experiment further validates the effectiveness of the proposed patch-TV algorithm. Under the circumstances of limited-view scanning in practice, patch-TV outperforms the two mainstream TV-based algorithms and is a practical and efficient reconstruction algorithm for PAI.

## Discussion and conclusion

In this paper, nonlocal patch regularization is incorporated into the TV-based photoacoustic imaging reconstruction model to effectively improve the performance in practical limited-view scanning. TV-based optimization minimizes the variation between adjacent pixels. It penalizes the local changes of the image and can therefore be referred to as local total variation. It is based on the assumption that the image is piecewise constant and over-suppresses the high-frequency coefficients. Thus, the geometric structure information of the reconstructed images tends to be over-smoothed. The result is even worse for practical limited-view scanning, in which the data information is insufficient such that serious artifacts and blurring fail to be effectively suppressed in the reconstructed images. However, in the nonlocal idea, the traditional spatial neighborhood is extended to the structured neighborhood in terms of geometric meaning, and the regularization is applied to patches in the whole image instead of only adjacent pixels [43]. Therefore, patch-TV shows great improvement in terms of the preservation of the images’ geometric structure and has better results in preclinical applications. The similar patches for weighted calculation for a certain patch Pxi are searched in the entire image according to the value of the weight function W(xi, xj). A threshold value T is set to screen the neighborhood of the patch Pxi. This method overcomes the problems in traditional nonlocal means (NLM) filters, in which the size of the search field is settled and the patch Pxi is estimated by the patches in the determined search field. Thus, for large areas, the calculation costs are increased rapidly, while for small areas, similar patches far apart are missed. Therefore, the size of the neighborhood of the patch Pxi is adaptively controlled. Moreover, the modified weight function is adopted in this paper. It utilizes the anisotropic distance between two patches to adaptively adjust the search of the neighborhood direction. For example, for edge points, their similar patches are searched along the edge direction. In this case, the neighborhood can be an ellipse. The neighborhood of the patches takes the directivities and geometric structure of the images fully into consideration. Therefore, this approach makes more reliable estimations for the weight calculations between patches. The application of this modified weighting calculation method, can better maintain structural and directional information of the images because of its more reliable estimation for the weights between patches. Furthermore, the optimization problem combining nonlocal patch and TV is simplified to a common iterative reconstruction problem. Thus, the solution process is significantly simplified. The variable splitting method and the Barzilai–Borwein-based method are adopted to further accelerate the calculation and convergence speeds.

The proposed patch-TV algorithm was validated by a series of simulations and an experiment. The simulations were conducted by means of straight-line scanning, which is often used in practical applications. The reconstructed results of patch-TV were compared to those of two mainstream TV-based algorithms: TV-GD and TV-Lp. The results show that patch-TV is superior to TV-GD and TV-Lp, whether judged visually or in terms of PSNRs. The artifacts caused by the data incompleteness are effectively suppressed, and the geometric structure of the images is well maintained. Furthermore, the noise robustness, the convergence and the calculation speed are also discussed. The experiment carried out on an in vitro phantom adopted traditional straight-line scanning with a single transducer. The results show that patch-TV outperforms the other two algorithms in each case, with more distinct geometric structure and fewer artifacts.

In this paper, the study is under a system-specific choice where the circumstance that laser pulses irradiate perpendicular to the image and not a result of having a 2D-reconstruction. While it is considered to be a common case which is easy to study. As for other cases, such as the light irradiate from other angles, we can use the Monte Carlo method in [66] to simulate the optical absorption distribution of the tissue. Actually, these cases mainly lead to the variation of the optical absorption distribution of the tissue yet the way to the algorithm study is the same.

The iteration number is set to 10 in this paper. As reported in [34, 39], the TV-GD and TV-Lp algorithm converged when the number of iterations is 10, which was an appropriate choice for these algorithms. Also as shown in “Convergence and calculation”, the line chart of the distance *d* in Fig. 8 confirms that the distance versus the iteration curve for these algorithms converges when the number of the iterations is 10, which validates the convergence of these algorithms at 10th iteration.

As for the parameter setting, *α* is the parameter corresponding to the weight of TV value in the optimization. *α* with a big value means that the TV-term is dominant and the optimization is expected to have a quicker convergence. But over-sized value will break the balance between the two parts of the objective function. The reconstructed images with over-sized *α* will have a great difference from the real images because the data fidelity in the reconstruction is sacrificed to the image regularity. Based on this criterion, *α* should be set to a value which is neither too large nor too small when compared with the weights of the other part of the objective function to ensure good reconstructions, noise robustness and convergence speed. *β* is the parameter corresponding to the weight of local-patch value in the optimization. It has similar effects on reconstructions, noise robustness and convergence speed to *α*. *T* is a threshold value ranging from 0 to 1 for screening the similar patches. Small value of *T* means that more patches with smaller similarities will be included into the neighborhood *δ*(***x***_*i*_) of ***x***_*i*_. It will diminish the effect of the constraint of local-patch and increase the time costs. While if *T* is set to an oversized value, few patches will be qualified for the neighborhood. So it may also degrade the performance of the algorithm. From the simulations and experiments, *α* can be set between 0.3 and 0.8, *β* can be set between 0.2 and 0.65, *T* can be set between 0.55 and 0.80.

It is also worth mentioning that the computation costs of patch-TV are higher than those of the other two algorithms due to the incorporation of nonlocal patch regularization. However, the quality of the images is significantly improved, and the convergence speed is greatly accelerated. Additionally, the simplification of the optimization problem and the utilization of variable splitting and the Barzilai–Borwein-based method make the solution efficient and fast.

As for the 3D extension, i.e. 3D PA tomography, the proposed patch-TV algorithm can be easily applied to it. The 3D PA tomography have the similar dataset and scanning mode with the 2D one. It’s also worth to mention that the patch-TV framework has space independent nature. The implementations can be fulfilled to 3D image reconstructions that use spatial information. But if we want to solving a 3D image volume, further studies need to be carried out. As we mentioned above, the whole converge time and single iteration time of the proposed patch-TV algorithm are just slightly more than TV-GD and TV-Lp algorithms, which makes the 3D reconstructions practical.

In conclusion, the proposed patch-TV algorithm is an effective and practical PAI reconstruction algorithm.

## Abbreviations

PAI: photoacoustic imaging
TV: total variation
TV-GD: gradient descent-based TV
TV-Lp: joint TV and Lp-norm
Patch-RE: the iterative algorithm only with patch-based regularization
PSNR: peak signal-to-noise ratio
FBP: filtered back-projection method
CS: compressed sensing
patch-TV: the combined nonlocal patch the TV regularization
SNR: signal-to-noise ratio
NLM: nonlocal means
